# Basic human values drive food choice decision-making in different food environments of Kenya and Tanzania

**DOI:** 10.1016/j.appet.2023.106620

**Published:** 2023-09-01

**Authors:** Christine E. Blake, Eva C. Monterrosa, Krystal K. Rampalli, Abdullah Nurus Salam Khan, Ligia I. Reyes, Shiny Deepika Drew, Paula Dominguez-Salas, Salome A. Bukachi, Mariah Ngutu, Edward A. Frongillo, Elyse Iruhiriye, Amy Webb Girard

**Affiliations:** a University of South Carolina, Arnold School of Public Health, Columbia, SC, 29208, USA; b Global Alliance for Improved Nutrition, Rue de Varembé 7, 1202, Geneva, Switzerland; c Cornell University, Division of Nutritional Sciences, Ithaca, NY, 14853, USA; d Natural Resources Institute, University of Greenwich, London, UK; e International Livestock Research Institute, Nairobi, Kenya; f Institute of Anthropology, Gender and African Studies (IAGAS), University of Nairobi, Kenya; g Rollins School of Public Health, Emory University, Atlanta, GA, USA

**Keywords:** Food choice, Food choice behavior, Drivers of food choice, Values, Basic human values, Food environments, Value negotiation, Kenya, Tanzania

## Abstract

Increased access to a variety of foods in low-and-middle-income countries (LMICs) has led to greater autonomy in food choice decision-making. Autonomy allows individuals to make decisions through negotiation of considerations in ways that are consistent with basic values. The aim of this study was to identify and describe how basic human values drive food choice in two diverse populations with transitioning food environments living in the neighboring East African countries of Kenya and Tanzania. Secondary data analysis was carried out on focus group discussions conducted with men and women in Kenya (n = 28) and Tanzania (n = 28) as part of prior studies on food choice. A priori coding based on Schwartz's theory of basic human values was conducted, followed by a narrative comparative analysis, which included review by original principal investigators. Values of conservation (security, conformity, tradition), openness to change (self-directed thought and action, stimulation, indulgence), self-enhancement (achievement, power, face), and self-transcendence (benevolence-dependability and -caring) were prominent drivers of food choice in both settings. Participants described how values were negotiated and highlighted existing tensions. For example, the value of tradition was cited as important in both settings but changing food contexts (e.g., new foods, diverse neighborhoods) increased prioritization of values like stimulation, indulgence, and self-directed thought and action. The application of a basic values framework was useful for understanding food choice in both settings. A focused understanding of how values drive food choice decision-making in the context of changing food availability in LMICs is essential for the promotion of sustainable healthy diets.

## 1 Background

Food choice is the process by which individuals and households decide what and how to produce, acquire, prepare, distribute, and consume food. Food choice occurs where individuals meet food environments and involves both conscious and unconscious decision-making (Blake et al., 2021; Sobal & Bisogni, 2009). Availability and accessibility are primary drivers of food choice in low-and middle-income countries (LMICs), especially for those struggling to meet basic subsistence needs (Gebremariam et al., 2017; Turner et al., 2020), but even under constrained conditions, what people value drives what foods they choose to eat and how they choose to eat them.

Increased access to a variety of foods and purchasing power in LMICs has led to greater choice and autonomy in food choice decision-making (Clark, Springmann, Hill, & Tilman, 2019). Autonomy allows individuals to act on goals and priorities that are consistent with their values. As food options increase, either becoming more available or more affordable, individuals and households modify food choice behaviors to fit with updated goals and priorities. Exposure to new foods or ways of eating provides opportunities to establish habits that can have positive or negative implications for health and environmental sustainability (Blake et al., 2021). Most populations that have experienced access to a wider variety of foods in markets and increased incomes also see diets that include more foods high in salt, sugar, and fat (Popkin, Adair, & Ng, 2012). A focused understanding of how values drive food choice decision-making in the context of changing food availability and accessibility is essential for the development of policies and programs that promote sustainable healthy diets.

In much of the food choice literature, food choice values are presented as considerations that people take into account when making food choices including cost, taste, convenience, and health (Connors, Bisogni, Sobal, & Devine, 2001). Cost, taste, convenience, and health, however, are attributes of food and not values. It is the values rooted in cultural context that shape how considerations are defined and negotiated. For example, what constitutes acceptable cost (e.g., price, value), desired taste (e.g., flavor, texture, preferences, social status), degree of convenience (e.g., time, access, packaging), or the relevant aspect of health driving decision making (e.g., absence of disease, management of a condition, child growth, community health, environmental health) is determined in part by deeper values. Understanding what these values are and how they are negotiated to influence what and how people eat would provide useful insights for the promotion of sustainable healthy diets.

In his foundational work on human values, Rokeach defined a value as “an enduring belief that a specific mode of conduct or end-state of existence is personally or socially preferable to an opposite or converse mode of conduct or end-state of existence” (Rokeach, 1973). His work demonstrates that prioritization of values partially accounts for variation in individual behaviors (Rokeach & Ball-Rokeach, 1989). Values represent a continuum of motivations and are ordered based on their degree of compatible or conflicting properties, orientation toward self-protection or self-growth, and whether they emphasize a personal or social good (Schwartz, 1992; Schwartz et al., 2012).

Values are prioritized in different situations to guide decision-making. Because values are grounded in three requirements of human existence namely, biological needs, social interaction, and survival of civilization, they are likely recognized in all societies (Schwartz, 1992; Schwartz et al., 2012). Schwartz proposed a theory of basic human values where values are defined as “*trans*-situational goals, varying in importance, that serve as guiding principles in the life of a person or group” (Schwartz et al., 2012). Values guide action and serve as standards or criteria for behavior (Schwartz, 1992; Schwartz et al., 2012).

Schwartz's theory of basic human values is applicable cross-culturally and has been tested extensively across settings, populations, and behavioral domains including health behaviors (Brümmer & Zander, 2020; Olsen, Atkin, & Thach, 2016; Puska, 2019; Spendrup, Eriksson, & Fernqvist, 2021; Thomson et al., 2017; Zander & Feucht, 2018). It has been applied to understand food and beverage choices among consumers, animal welfare considerations for beef choice, regulatory focus in promotion of domestic food products, and genetically modified food (Botonaki & Mattas, 2010; Bryla, 2021; Dreezens, Martijn, Tenbült, Kok, & De Vries, 2005; Sonoda, Oishi, Chomei, & Hirooka, 2018; Yang et al., 2019). To date, most applications of the Schwartz theory of basic human values to food choice have been in upper-middle-income or high-income countries.

Understanding whether and how basic human values drive food choice in LMICs is important for the proactive design of policies, programs, and messages that result in demand for and adoption of sustainable, healthy diets among populations experiencing rapidly changing food environments (Clark et al., 2019; Turner et al., 2020). Evidence for basic human values as drivers of food choice exists for high-income countries but is lacking in LMICs, especially in the context of changing food environments. In LMICs, the increasing availability and demand for ultra-processed foods that are low in nutrients (Vandevijvere et al., 2019), especially among children and youth (Maxfield, Patil, & Cunningham, 2016), is concerning due to rising rates of overweight and obesity and non-communicable diseases (Irache, Gill, & Caleyachetty, 2022; Popkin, Corvalan, & Grummer-Strawn, 2020). Similarly, widespread consumption of diets high in refined carbohydrates, saturated or trans fats, and sodium across the world including in LMICs is alarming (Cairns, Angus, Hastings, & Caraher, 2013; Ludwig, Hu, Tappy, & Brand-Miller, 2018). Such changes in diet are contributing to rising rates of overweight and obesity in many countries in Africa, including Kenya and Tanzania (World Health Organization, 2021). A recent cross-sectional community-based study in Dar es Salam, Tanzania found very high rates of overweight (35%), obesity (32%), and morbid obesity (9%) in a random convenience sample of 6691 adults shopping in a large urban market. Authors note multiple self-reported diet related non-communicable diseases and the burden that these place on already strained health systems (Pallangyo et al., 2020). Understanding drivers of food choice behavior that contribute to obesity in LMIC contexts is imperative to addressing the growing burden of diet related NCDs.

The aim of this study was to identify and describe how basic human values drive food choice in two diverse populations with transitioning food environments living in the neighboring East African countries of Kenya and Tanzania. These two settings offer insight into how values undergird food choices in the context of changing food environments and livelihoods that increase consumers’ availability and accessibility to unhealthy, ultra-processed foods. Such knowledge will be useful in designing contextually appropriate social messaging, programs, and policies to promote sustainable healthy diets.

## 2 Methods

### 2.1 Study overview

Secondary analysis was performed on data from two studies, one conducted in Kenya and the other in Tanzania. These studies were part of the Drivers of Food Choice Competitive Grants Program, which aimed to explore drivers of food choice among the poor in the context of changing food environments of countries in sub-Saharan Africa and Asia (Blake et al., 2021). The two studies collected data using similar methods and interview guide questions. Kenya and Tanzania are in the Eastern Africa Region and share similarities in both culture and dietary practices, but each study population was at a different stage of development and nutrition transition with differing food environments (Bukachi et al., 2021; Ripkey et al., 2021). The Kenya study examined supply and demand drivers for animal source foods (ASF) among low-income residents of urban informal settlements. The Tanzania study explored how the process of rapid sedentarization (e.g., the settling down of previously nomadic groups) among traditionally pastoral communities contributed to changes in food choice. The two samples allowed for examination of the range of values driving food choices in populations with some similar cultural traditions but different food environments.

### 2.2 Data sources

The qualitative data from Kenya study were derived from a sample of participants residing in Dagoretti North and South sub-counties, urban informal settings in Kenya's capital, Nairobi City County. The sample population has a high density of livestock ownership. A total of 38 focus-group discussions (FGDs) were conducted with couples living in the same household and having at least one child under age five (Bukachi et al., 2021; Dominguez-Salas et al., 2016). Of these, 28 FGDs that included detailed description of values driving food choice were selected for analysis. The FGDs were conducted in Kiswahili and separated by sex, with 12 men's groups and 16 women's groups. Each FGD included an average of 12 participants, conducted over the course of two half-days to minimize burden on participants given the density of the FGD guide (Table 1).

**Table 1:** Focus group discussion participants by location, gender, and life stage.

	Kenya (a)	Tanzania	Total
*Gender*			
Men	12 (144)	12 (96)	24 (240)
Women	16 (192)	16 (128)	32 (320)

*Life stage*			
Youth		10 (80)	10 (80)
Adult	28 (336)	9 (72)	37 (408)
Elders		9 (72)	9 (72)
**Total Focus Groups (# participants)**	28 (336)	28 (224)	**54 (560)**

a Each set of men and women participated in two FGDs.

The Tanzanian study was conducted in six villages in rural Handeni and Mvomero districts. These villages were purposively selected from a larger sample of 30 villages participating in a dairy development project implemented by the International Livestock Research Institute. Village selection was based on diversity in predominant livelihood strategy, levels of sedentarization, proximity to a market, and demographic characteristics (Ripkey et al., 2021). A total of 54 FGDs were conducted across two seasons in these six villages. FGDs were stratified by age, tribe, and gender. Of these, 28 FGDs included detailed discussions about values driving food choice and were selected for analysis. Each focus group included an average of 8 participants (Table 1).

FGD guides for both studies included questions about food choice behaviors and decision-making, including values driving these behaviors (Table 2). Both studies generated detailed data on food choice behaviors and drivers of those behaviors from the perspective of participants that elicited responses about values driving food choice. Elicitation of basic human values undergirding food choice allows for contrasting and comparing those values across contexts. Both studies received institutional and country approvals for human subject research. The Tanzania study received ethics approval from Emory University Institutional Review Board (Atlanta, USA), Sokoine University (Morogoro, Tanzania), and the Tanzania Ministry of Health. The Kenya study received ethics approval from the London School of Hygiene and Tropical Medicine Research Ethics Committee (London, UK) and the International Livestock Research Institute Research Ethics Committee (Nairobi, Kenya).

**Table 2:** Sample questions from focus group discussion guide for both parent studies.

Kenya
• What are some of the animal source foods (ASF) that are commonly consumed in your household? Why? Probe (*where it is accessed from; Is it healthy, easy to get, tasty or easy to prepare*). • Do you have any access to own produced food? If so, what and from where? How important is it in your total food supply? • Particularly, when people have animals, is it mostly used for household consumption or for sale? Who decides that? If sold, who decides how to allocate the money? Are they mostly in the city or are they upcountry? How does the family benefit from them if the animals are upcountry? • What are the key factors that affect the access to ASF in this community? • Who decides what to eat? How is that communicated to other people involved? o Who is responsible for buying foods from the market? oHow does your family decide on how much money to spend on ASF? How does the income level of the household head impact on the household security? oWho is involved in the decision? Who has the final say? In your opinion, who do you think should have the final say? oAre there any gender differences in the food taboos in your community? • How strictly are they observed nowadays? Is it culturally acceptable to ignore that? Are there any voices confronting any of these taboos?
**Tanzania**
• Why are foods in [*Insert name of first food/group*] commonly eaten in this community? • How does the consumption of foods in *[insert name of first food/group]* change seasonally? oWhere do you usually get this food? ⁃ Probe: in the market, domestic consumption, wild, bartering, other ways? • Which wild foods are commonly eaten in this community? [as they are named, list on the flip chart then probe] • How is this food important in your community? oProbe: Is it culturally important to your tribe? oProbe: Is it eaten on special occasions? • Why are foods in [*Insert name of first food/group*] rarely eaten in this community? oAre any of these foods taboo? If so, why? oIf you could eat any of these foods, would you? If so, which ones and why? oWhat barriers prevent you from eating this food? ⁃ Probe: Why? oWhat does this food make you think or feel? Why? oYou have put (participant food list) as easiest for most people to eat more often. How often do people eat these foods? oWhere do people get these foods? oWhy did you put these foods as being the easiest for most people to eat? ⁃ Probe on availability/seasonality, price, acceptability oWhich people might not find these foods easy to eat more of? ⁃ Probe on gender, age, (older vs. children), poverty, etc.

### 2.3 Data management

FGDs in both settings were audio recorded, transcribed verbatim, de-identified, and translated to English. De-identified transcripts were provided to the authors using a secure file-sharing service. The transcripts were uploaded into Nvivo 12 software for analysis (QSR International Pty Ltd, 2018). Original Word files of the transcripts and the Nvivo 12 project file were stored in password-protected folders, only accessible by study personnel.

### 2.4 Development of the codebook

A codebook was developed using an iterative process. The initial codebook drew primarily on Schwartz’ basic human values, which consist of four high order domains (Table 3) and then specific values under these domains (Schwartz et al., 2012). The codebook definitions were further informed by Hofstede's Cultural Dimensions Theory (Hofstede, 2011) to include other possible relevant values driving food choice in these contexts (Table 3). This initial codebook was refined through double coding of a small subset of FGDs from Tanzania (∼10%) using line-by-line coding with Nvivo 12.

**Table 3:** Final codebook (adapted from Schwartz et al., 2012).

Higher order values or domains	Basic values code	Description or conceptual definitions in terms of motivational goals
Conservation		Self-restriction, order, avoiding change
	**Security-personal and societal**	Security of self or those with whom one identifies; safety in one's immediate environment and/or safety, harmony, and stability in the wider society (national security, social order/societal stability). This includes maintaining or ensuring food security in household
	**Conformity-rules and interpersonal***(Merged with*Hofstede's (2011)*restraint value)*	Restraint of actions or inclinations likely to violate social expectations and formal obligations (e.g., self-discipline, resist temptation) and/or restraining actions to avoid upsetting others or harming other people (politeness, honor parents, show respect)
	**Tradition**	Respect, commitment, maintaining, and preserving the customs and ideas that one's culture or religion provides
	Humility*Humility fits in both the higher order values of conservation and self-transcendence	Renunciation of self-interest; expressions of humbleness, modesty, not asking more for self, not drawing attention to self and/or recognizing one's insignificance in the larger scheme of things
Openness to change		Being accepting of change in a variety of context and situations
	**Self-direction- thought and action**	Emphasis on freedom and independence of thought and action. Independent thought comes from needs for control and mastery and/or freedom to determine one's own actions and to attain self-chosen goals
	**Stimulation**	Prioritizing excitement, newness, or change to achieve from the organismic need for variety to achieve an optimal, positive experience.
	**Indulgence***[Merger of*Hofstede's (2011)*indulgence value and*Schwartz et al.'s (2012)*hedonism]*	Seeking gratification of human desires to enjoy life and having fun. This might be manifested as people purchasing specific food items as an occasionally ‘treat’ rather than a regular occurrence.
Self-enhancement		Pursuing one's own interest
	**Achievement**	Success according to social standards; the underlying motivation here is to be judged successful by others (performance motivation). Achievement values emphasize demonstrating competence in terms of prevailing cultural standards, thereby obtaining social approval
	**Power-dominance and resources**	Power through exercising control over people; emphasizes the attainment or preservation of a dominant position within the more general social system and/or power through control of material and social resources
	Face*Face fits in both the higher order values of self-enhancement and conservation	Maintaining prestige, security, and power through maintaining one's public image and avoiding humiliation
Self-transcendence		Transcend one's own interests for the sake of others
	**Benevolence-dependability**	Preserving and enhancing the welfare of those with whom one is in frequent contact with the in-group: Being a reliable and trustworthy member of the in-group (dependable, loyal/faithful to friends)
	**Benevolence-caring**	Benevolence values emphasize voluntary concern for others' welfare; devotion to the welfare of in-group members (helpful, working for others' welfare)
	**Universalism** -**societal concern** -nature -tolerance	-Understanding, protection, and commitment to equality, justice, and protection for all people -Preservation of the natural environment because failure to protect the natural environment will lead to destruction (unity with nature, world beauty) Acceptance and understanding of those who are different from oneself (broad-minded/tolerant, mature, understanding)

Throughout this first coding process, three authors from the Kenya project team were consulted to ensure accurate interpretation of coded passages. This led to further modifications of the codebook. For example, upon discussion with the local Kenyan researchers, it was determined that the Schwartz value of hedonism was not an appropriate characterization of the food culture and food choices made among either of these East African study populations. Rather, a similar activity of seeking and valuing pleasure through occasional consumption of certain foods could be considered an “indulgence,” as previously described by Hofstede (Hofstede, 2011).

Several similar human values codes were merged into single thematic categories (Table 3) including self-direction (thought and action), power (dominance and resources), security (personal and societal), and conformity (rules and interpersonal). Food choice considerations (e.g., taste, health, cost, convenience) and food choice behaviors (e.g., food preparation method, vendor choice, storage) were also included based on food choice literature and used to describe how values drive food choice decision-making in these settings.

### 2.5 Data analysis

The analysis was organized in three steps, first to describe whether the basic human values were expressed in food choice decision making, second to explain how each value was expressed in food choice decision making, and third to compare and contrast the expression of basic human values across these two contexts and across sex and age groups where possible. Using the codebook developed for this study, we conducted deductive *a priori* coding using the constant comparative method (Glaser & Strauss, 1967). This method entailed coding various passages (units) and putting into categories (values and higher order domains), and then revising and redefining relationships between values and domains, food choice behaviors, and food choice considerations throughout the process. The FGD transcripts were coded by four coders (EI, KR, AK, and LR) to identify the basic human values associated with passages describing a food choice behavior. The Kenya transcripts were each read and summarized in a brief narrative using themes from the codebook that highlighted the key values expressed, key food choice considerations, and additional contextual comments. The Tanzania FGD transcripts were then coded and summarized in brief narratives with supporting quotations that highlighted the key values expressed, key food choice considerations, and additional contextual comments.

The description of how higher order human values (e.g., conservation, self-enhancement) influenced food choice decision-making (e.g., purchasing, consuming) was compared by setting (Kenya versus Tanzania) using the brief narratives.

Steps were taken throughout the research process to ensure quality (Corbin & Strauss, 2007; Miles & Huberman, 1994; Whittemore, Chase, & Mandle, 2001). The team engaged in regular peer review at all steps of the process, including involvement of the principal investigators of the Kenya (PDS) and Tanzania team (AWG) in an iterative peer review that involved at least quarterly meetings before and during analysis. An audit trail with records of the coding process and decisions made at each step was used.

## 3 Results

Values from all four domains of Schwartz's theory of basic human values (i.e., conservation, openness to change, self-enhancement, and self-transcendence) were evident in participants' descriptions of food choice decision-making. Evidence of how each value influenced food choice is presented by domain. Prioritization of values across domains, sub-groups, and settings follow.

### 3.1 Conservation domain

The conservation domain comprises security-personal values, conformity-rules, tradition, and humility. Humility was not reflected as a value driving food choice by participants in this study. A summary of each of the conservation values that guided food choice decision-making is presented below and in Table 4.

**Table 4:** Expression of values through food choice behaviors (**Conservation domain**).

**SECURITY-PERSONAL**
***Kenya***
• Health and preference of the children and household head influence the food-related decision-making in the family • Price of food is often prioritized over the nutritious value • Amongst the uncertainty of food contamination in the market, previous experience with known food vendors is highly valued • Rearing livestock is commonly practiced ensuring food and financial security of the household
***Tanzania***
• Ensuring food availability in the household is crucial to protect the well-being of the household members • Ideas of security as a food choice value differ between men and women
**CONFORMITY-RULES**
***Kenya***
• Taboos and social norms of different ethnic communities restrain/forbid people from consuming certain animals or animal parts/products • In contrast to women and children, the male head of household has a higher social value ascribed to him • Pregnancy is considered a special state of women when not all foods are allowed for consumption
***Tanzania***
• Social norms unexplainedly allocate certain foods to specific members of the household • Youth are strongly in favor of changing food-related taboos and myths
**TRADITION**
***Kenya***
• Serving meat during social events is considered prestigious • Serving different types of meat to different family members symbolizes the social values ascribed to them • Exchanging animals is accepted as a form of dowry payment • Ethnic-specific traditions have close relation with food-related beliefs, choices, and behaviors • Although not consumed in some cultures, certain animals/animal parts are treated sacred
***Tanzania***
• Traditional practices are inherent in food-related beliefs and practices • Modernization affects many of the traditional practices, especially among the youth

#### 3.1.1 Security-personal

Participants in Kenya and Tanzania valued the safety and security of their households as caring for the health of family members and seeking food security. When food was scarce, food security was valued over other general health concerns. The health needs of elders and younger children were often prioritized over other household members. Mothers sought nutritional quality for child health. Food safety was a major concern among Kenyan participants as they had reported contamination of milk and eggs – foods that children commonly consumed. Participants’ perceptions of food safety meant they purchased food from sources they trust:

*“… the informal vendors … it has many issues. Maybe the containers that it (milk) is put in are not clean, or the person measuring the quantity is using things that are not clean, or the people milking it are adding water to it, sometimes they add blue band [margarine] or even powder milk.” (FGD_Women_Kenya)*

In both Kenya and Tanzania, livelihoods traditionally depended on pastoralism for both financial and food security as livestock served as both food and income sources:

*“it is good to have livestock in your homestead, at the same time it is food … The advantage is when you keep it you can get milk out of it and in future you can sell and it can be meat. Majority of them [livestock] have advantages … And still that milk you can sell it and you can use it at home” (FGD_Women_Kenya)*

While women valued economy for food security when making decisions in the marketplace, one woman suggested that men's food choice behaviors may not be as strongly influenced as women's by concern for food security. Participants described the deleterious effects of climate change on livestock viability and agricultural productivity as a reason for some of the dietary shifts and livelihood changes. Changing livelihoods have altered gender roles in some households thus making both husband and wife active participants in ensuring food security:

*“There are great changes because, in the past years, men depended on the availability of milk, so women were the ones who were supposed to go to the market and buy food varieties. But nowadays men have become more responsible to ensure the availability of food in their households due to life changes.” (FGD_Men_Tanzania)*

#### 3.1.2 Conformity-rules

Values of tradition were closely intertwined with conformity-rules. In both Kenya and Tanzania, social expectations and formal obligations existed. Both communities had certain tribe-specific traditions and practices led by elders. Across gender and age, rules were followed without questioning.

Taboos are social customs, or rules, prohibiting a particular practice and often are related to negative consequences. Certain animal parts were considered taboo. Some taboos described as important by Kenyan participants included:

*“When you are pregnant, you are not advised to eat eggs so much … It has a lot of proteins and at times they say that the eggs will make the baby very big” (FGD_Women_Kenya)*

*“It is said that if you give them (eggs to the children) early they will delay in speech development … They will have a heavy tongue.”* (FGD_Women_Kenya)

*“In my community chicken is commonly eaten, but when you get married your husband can’t slaughter a chicken for you … (it is) against our customs … they believe that when he slaughters the chicken for you, your marriage will not hold, you will separate …” (FGD_Women_Kenya)*

Tanzanian participants described taboos based on beliefs about how present actions might affect future consequences. For example:

“*It was taboo that a woman cannot finish all the milk. It was believed that if she does so all the family herd can die.” (FGD_Women_Tanzania)*

Valuing conformity to rules influenced how food was allocated to specific members of the household. In most cases, women and children were restricted from consuming certain animal parts out of respect to men:

*“In my place, if we slaughter a chicken, we are not supposed to give the man of the home the bone or the back, you are supposed to give him the thigh or the leg of the chicken. Someone like me cannot serve the children, the thigh and give the husband a back that is not respect.” (FGD_Women_Kenya)*

#### 3.1.3 Tradition

In both Kenya and Tanzania, food traditions were upheld in familial gatherings, cultural events, and religious celebrations. Conformity to tradition was related to food taboos both prescriptive and proscriptive, however, participants from both communities were inclined to refrain from conforming to some traditions. Both communities sacrificed animals to celebrate social or religious events. Many of the food customs were reflected as gender norms. According to some participants, erosion of the value of tradition coincided with modernization of society in Tanzania.

In Kenya, animal source foods (ASF) played a key role in cultural traditions. For example, chicken is slaughtered to impress guests at home gatherings especially among the *Abaluhya* community. Larger animals such as goats and cows are slaughtered in socially important events like funerals and bride price ceremonies, rather than smaller animals like chickens which are slaughtered mainly for routine family level occasions:

*“It is the Agikuyu custom that a goat has to be slaughtered … for the mother who has given birth to recover and be strong. So, when I give birth in the Agikuyu community there was a guarantee of eating goat.” (FGD_Women_Kenya)*

Tradition dictated how different animal parts are consumed by different family members to show respect:

*“It shows that the man was the head of the homestead; he was important because he was the owner of the homestead. So, (a cow) has to be slaughtered as a sign of respect.” (FGD_Women_Kenya)*

In some Kenyan communities, the male head of the household receives meat from the thigh, back, and shoulder, whereas the women and children get meat from the head and feet of the animal. In Tanzania, traditionally, men refrain from consuming meat from an animal that has died in the presence of a woman. A male participant from Kenya mentioned his thoughts about this tradition:

*“… about tradition also, among the Luo, women are not allowed to eat gizzards. That is food for old men. They are not supposed to eat. I do not know if that was a way to oppress the women because like children they are told their foods are legs and heads so that when they eat they become clever”*

*(FGD_Men_Kenya)*

Specific traditions are upheld at cultural events, such as at a circumcision ceremony,

*“In initiation ceremonies, the uncircumcised boys are given the intestines and the rest of the meat is for the men including those who have been recently circumcised.” (FGD_Women_Kenya)*

Traditional spiritual beliefs influenced food choices. For example, lard is used in certain communities to ward off what was termed as “bad eyes,” defined as evil intentions of certain people. Religious traditions also restricted individuals from consuming specific foods:

*“… when it comes to religion, you will get like the seventh day Adventists; they are not allowed to eat any animal that does not have hooves, isn’t it, or the one that does not chew cud. Then about fish, they are not allowed to eat fish that does not have scales … In Luo, we call them ‘okoko’, those do not have scales, so they are not supposed to eat them (okoko fish).” (FGD_Men_KENYA)*

Traditions were a source of tension between the youth and elders. Adult and elders expressed dissatisfaction at young people abandoning food-related traditions,

*“Yes, it’s true in the past we used to drink animal blood as part of our daily life and this was mostly due to culture and beliefs, it was a belief that animal blood helps to increase blood to the pregnancy women also it was used during spiritual ceremonies as a sacrifice to those who have problems or who need blessings, but now we no longer believe that, so we don’t drink animal blood.” (FGD_ Elder Women_Tanzania)*

Elderly participants in Tanzania attributed the decline in blood consumption to several factors including formal schooling, the spread of Christianity into the communities:

*“Education and religion more especially Christianity, many people have gone to school and become educated, still others have become Christians and been baptized, so they live according to the bible and church preaching which are against unnecessary customs and norms. For instance, they stopped drinking cow blood and using “vibuyu” (traditional calabash) to drink milk.“(FGD_Young Women_Tanzania)*

Globalization and modernization were also cited as explanations for the reduction in consumption of blood:

*“Changes in diet have affected the youths because in the past years most youths used to eat foods at homes but nowadays, they just eat foods at the food vendors.”*

*(FGD_ Young Women_Tanzania)*

*“Changes in diet have affected our communities/tribes because many people have abandoned natural and traditional food stuffs, these changes have created an image which did not exist before.” (FGD_Young Women_Tanzania)*

### 3.2 Openness to change domain

The domain of openness to change contains the values of self-direction (thought and action), stimulation, and indulgence. Food choice was influenced by self-direction and stimulation in both Kenya and Tanzania. In contrast, indulgence was only mentioned by participants in the Kenya study (Table 5).

**Table 5:** Expression of values through food choice behaviors (**Openness to change domain**).

**SELF-DIRECTION (THOUGHT AND ACTION)**
**Kenya**
• Loosen traditional prohibitions for food consumption • Maternal autonomy in making child-feeding decisions • Maternal strategizing to make foods safe and digestible for child • Maternal-driven decisions about foods in family meal
**Tanzania**
• Women's freedom to bring home the foods that she desires • Women's agency to spend their earnings how they see fit • Seek benefits observed in others by changing subsistence practices
**STIMULATION**
**Kenya**
• Embrace diverse cultures and introduction of new foods • Shift preferences for new foods from tasting opportunities • Seek variety to break monotony in food consumption
**Tanzania**
• Break monotony of traditional diets by trying new foods and preparation methods found to be appetizing • Continue food practices that were brought upon by life circumstances but found to be enjoyable • Eat in restaurants to try new foods and break monotony in diets
**INDULGENCE**
**Kenya**
• Rejoice in eating foods that bring pleasure to life • Purchase meat for emotional satisfaction • Incorporate meats in celebratory meals • Allow family and visitors to enjoy meat on rest days as reward for hard work

#### 3.2.1 Self-direction (thought and action)

Self-directed thought and action are defined as values that emphasize freedom to independently cultivate one's ideas and actions originating from self-control and mastery. These were expressed as independent decision-making. Women described valuing self-direction over food budget management, food purchasing decisions, and meal preparation:

*“… speaking on my own behalf, it is the wife. I am the one who decides what I want to do, neither the husband nor the children. Husband is someone who comes and I give him food … he is supposed to eat that time and I am the one in charge of the timetable, what to cook today or not.” FGD_Women_Kenya)*

In both Kenya and Tanzania, women took charge of meeting their children's nutritional needs. The following passage explains how a woman from Tanzania exercised self-direction in spending money:

*“As for me as I have my children; if the child has a problem, I can help with the money I have so as that child can solve the problem. However, it is not mandatory. With me I look for my money, I get it, I spend it the way I want and solve my problems and invest in whatever I think I can. I can buy livestock drugs, I can buy food, clothes or whatever is needed in my home. So, we complement each other. The husband fends for the house but I can also help” (FGD_ Elder Women_Tanzania)*

#### 3.2.2 Stimulation

In both Kenya and Tanzania, increasing opportunities to taste different foods led to individuals value of stimulation as a driver of food choice:

*“Interviewer: Why do you eat rice a lot nowadays?*

*Participant: When we go to the Centers and eat at a food point; we can ask for rice or any other food and when we eat we find it good and tasty and this attracts us to prepare the dish ourselves when we are at home. Also at the markets these dishes are sold and so you can buy the rice and cook it at home.” FGD_ Adult Women_Tanzania)*

Participants described monotony in their daily diets and the importance of variety and change:

*“The staple food for Manyinga community is rice. You prepare rice and stiff porridge interchangeably. Old women may get tired of the same dish everyday … so they want to change diets.” (FGD_Young Women_ Tanzania)*

Also, mothers were especially attuned to children's food preferences,

*“If children do not like a certain type of food they will tell you and you will have to cook a different type of food of their favorite.” (FGD_Young Women_Tanzania)*

In both Kenya and Tanzania, commingling of various ethnic groups resulted in the adoption of newer, more palatable foods:

*“We learned how to cook these other dishes gradually. We can even cook Amaranth greens very well … It was available [in the past]. But we did not know that it was a delicious and palatable dish. But, our neighbors, who are of other tribes and friendly, started welcoming us and made us taste some of the dishes they were making, and we saw that they were delicious. So, we started adapting the trend and started learning to cook the other types of food and here we are” (FGD_Adult Women_Tanzania)*

#### 3.2.3 Indulgence

The value of indulgence as a driver of food choice was observed only in Kenya. Meat was purchased for emotional satisfaction and as an indulgent purchase for celebratory events and for serving visitors. Participants talked about the value of indulging in expensive food items as a reward for performing hard work or to make their children happy:

*“… meat is something you will buy only when your heart wants or when the children want” (FGD_Women_Kenya)*

Some described valuing indulgence in food choice decisions as a way of life, recognizing that the future is not predictable:

*“We do not know about tomorrow so we eat and enjoy life. You eat well because you do not know about tomorrow. You can get out of here and be knocked by a motorbike yet you have hidden money under the mattress. So you eat well” (FGD_Women_Kenya)*

### 3.3 Self-enhancement domain

The self-enhancement domain comprises values of achievement, face, and power (dominance and resources) (Table 6).

**Table 6:** Expression of values through food choice behaviors (**Self-enhancement domain**).

**ACHIEVEMENT**
***Kenya***
• Owning livestock as a concrete demonstration of wealth/success that others can judge • Eating certain foods, particularly ASF, as an indicator of wealth/success • Serving certain foods, particularly ASF, to visitors/guests or for special occasions
***Tanzania***
• Foods purchased were valued more than foods farmed
**FACE**
**Kenya**
• ASF considered necessary to serve to guests or at events, or others would think poorly of you • Having a strictly vegetarian meal for guests would be insulting •
**Tanzania**
• Adoption of fishing as a quick income-generation activity even though it is looked upon poorly by others • Valuation of specific foods due to cost or another prized characteristic • Preservation of status since pastoralism was considered “non-fashionable” • Eating certain foods to impress people, even if unhealthier
**POWER (DOMINANCE AND RESOURCES)**
***Kenya***
• Individual who has the money is the one that makes food choice decisions for the household
***Tanzania***
• Valuation of youth getting more education and leaving pastoralist livelihoods, moving to cities, and making more money • Conflicts with local farmers when pastoralists migrate from one area to another with their livestock

#### 3.3.1 Achievement

Owning livestock was considered a sign of achievement and symbolic of wealth and success. In Tanzania, ownership of cattle signified wealth, prestige, and social status:

“*Generally you will get that in terms of wealth accumulation, it has significance. For example, you can go somewhere and you get so many goats and cattle. This can symbolize that such a family is stable or well off, .… While you can go to another place where there are no cattle, chicken or anything. So that is the perception of people. It symbolizes that when you have many cattle, many goats, at least you are well off.” (FGD_Adult Men_Tanzania)*

Participants in both settings equated the social status of a family with the cost of the food items they consumed. Many participants explained that social status can be obtained by having others see them eating certain foods because these foods are indicative of how much money one has or one's social class.

*“… something like fish, chicken … You are told you are of high class because you are able to reach that price, and there is this ‘katakata’ which is easily available, you get those for KES 30, there is single, double patry which is for the rich, but most of us are at katakata level.” (FGD_Adult Men_Tanzania)*

*“In the case of unprocessed maize meal “dona”, the price of this type of maize meal was lower than the processed maize. Hence, when a person finds that you are using that type of maize they will also rate you as of low class. So, people were trying to impress people rather than caring about their health. So, they see it better to use the processed food which actually does not have many nutrients.” (*FGD_Young men_Tanzania)

Rural pastoralist Tanzanians in this study did not identify consumption of ASF as a marker of social status in the same way as peri-urban Kenyans did, because all pastoralists had access to some ASF. In both Kenya and Tanzania, even when they expressed a desire to eat healthier or had knowledge about healthier foods, people stated that they would purchase ultra-processed or unhealthy foods to serve and show others that they can afford to feed their families with more “fashionable” foods. This value of achievement was also prioritized to justify more expensive purchases. Similarly, food purchased were preferred over collected wild ones:

*“When we were still young, we used to spend a whole day in the wild collecting fruits such as guavas, playing different child games and we come back in the evening, but nowadays we don’t depend on those fruits. The wild fruits are available but nowadays we have valuable fruits such as mangoes, pineapples, ripe bananas, etc.”* (FGD_ Adult Men_Tanzania)

#### 3.3.2 Face

In Tanzania, most pastoralists appeared to be less concerned with face as most of them shared a similar socio-economic situation. In Kenya, participants spoke extensively about how they treated visitors and what foods were served to them. ASF were considered mandatory to serve guests or provide at events. Participants stated that if ASF were not provided, then it would be a poor reflection on them. It was also widely acknowledged among participants that vegetables were healthy to consume but, having a strictly vegetarian meal was not ideal and would not impress outsiders.

“Interviewer: Okay so without chicken there is no event?

Participant#1: Yes.

Participant#2: If you go visiting [for example, we visit you], … and you cook for us vegetables without meat or chicken it is like you have disrespected us.

(FGD_Women_Kenya)

Participant#5**:** Even when somebody eats vegetables, it is like he is torturing himself, so he reckons he needs to feel good therefore he adds meat to his meal.

Participant#9: In fact, vegetables on a plate without meat look bad.

(FGD_Men_Kenya)

#### 3.3.3 Power (dominance and resources)

In both Kenya and Tanzania, power was highly valued and was expressed through showing dominance and resources. Power was manifested in the form of money, livestock, or possession of intangible resources such as education in both settings. In Kenya, money was the indicator for determination of one's power in the household and in the community. Therefore, in these communities, it was common to see women having more independence and ability to control the food choice decision-making for the household:

*“Interviewer: Alright. Now if we go back to the home and we consider the issue of making a decision on what will be cooked, or what will not be cooked. Who in particular makes that decision in your home? If it is meat, goat, vegetables.*

*Participant: Whoever has money.*

*Interviewer: Who is this that has money?*

*Participant: Whoever has money is the one who will decide what food will be cooked.*

*Interviewer: So if it is the head of the house who provides the money, then he decides or if it is the woman, then she decides.*

*Participant: Yes, because the woman of the house cannot say that she wants meat while you don’t have enough money to buy it but have a little to buy ‘omena’ or ‘katakata’.” (FGD_Men_Kenya)*

In contrast, in Tanzania, livelihood was seen as a source of power, with farmers wielding more power than pastoralists:

*“In the community there are three main cadres. You have the high class, low class and the middle which carries the most population. So what is the difference as far as food is concerned focusing on the low and high class. That of high class, getting chips or chicken that is well made is normal. But this one who is in the middle class …. … the one who is low at the most he can eat with Mlenda, unprocessed maize meal known as “dona”, or cassava meal, when he changes he used Amaranth green vegetables or dagaa. A person in high class eats dagaa but this can be something he eats as a change of diet and not the only thing that he is able to afford to sustain his life. The one in low class can use the same diet for weeks on end. And the one in high class will prepare the dagaa well and even order those from far which are more tasty and expensive. The dagaa prepared by the one who has high class is prepared more elaborately with a variety of spices while the one of low class are just boiled and put a minimal amount of spices or none at all just boiling plus a little oil and salt.”*

*(FGD_Young Men_Tanzania)*

Some pastoralists saw education as an important step to providing children an alternative lifestyle to pastoralism, which had implications for food choice. One woman explained how their children going to school impacted their diet practices:

*“In the past, people were not educated. So, after going to school when they come back they transform the environment … (in the schools) they were staying in dormitories, they were eating beans, fish, small dried sardines; but such things were not available here. So, when they come here they usually have the urge to use the things they were using in school. Actually, they transfer the behavior and style they were used to in school and bring it here. That changes the diet trend in that they leave what they were used to use here and are now using what they were using in the town.” (FGD_Young Women_Tanzania)*

### 3.4 Self-transcendence domain

The self-transcendence domain comprises values of benevolence (caring and dependability), humility, and universalism (tolerance, nature, and concern). Only the value of benevolence was relevant for guiding food choice decision-making (Table 7).

**Table 7:** Expression of values through food choice behaviors (**Self-transcendence domain**).

**BENEVOLENCE-CARING**
***Kenya***
• Provide ASF to enhance children's development • Forego adult ASF consumption to prioritize children • Bring joy to family through food • Acquire and prepare foods that respond to family preferences
***Tanzania***
• Forego/scale-back adult ASF consumption to prioritize children • Provide ASF to enhance children's development • Prioritize serving meal to family before self (women) • Bring joy to children through food
**BENEVOLENCE-DEPENDABILITY**
***Kenya***
• Seek trustworthy and reliable vendors • Convey loyalty as customers • Establish criteria for perceived food safety
***Tanzania***
• Find means (men) to feed family, especially children • Rely on neighbor support during food shortages

#### 3.4.1 Benevolence-caring

In both Kenya and Tanzania, wellbeing of children and the prioritization of their diets was a prominent expression of the value of benevolence-caring. Parents explained that they provided ASF to their children to demonstrate their love and care:

*“You slaughter sheep or goat for your school kids so as to show them love and care and create happiness for them.” (FGD_ Adult Women_Tanzania)*

Another common expression of this value, especially among women, was the importance of putting the family before the self in decisions about what foods would be prepared. In Kenya this was expressed as being responsive to family food preferences or shared decision-making with spouses. In Tanzania, it was demonstrated through a gendered expression of care, with women being selfless. For example, women described serving their spouses and children before serving themselves *or* foregoing their own consumption of prized ASF to ensure that children consumed these products with sufficient frequency, especially milk.

#### 3.4.2 Benevolence-dependability

The value of benevolence as dependability was described primarily in relation to food vendors and relationships within communities. In Kenya, the meat vendor and purchaser relationship were built on dependability and trust. In the consumer-vendor relationship, dependability included vendor cleanliness, service quality (e.g., customer treatment), and extending food on credit. Having a preferred vendor meant that participants could trust the quality of the meat purchased, and even when there was an issue, there was comfort in knowing that this would be addressed.

*“For example, if I buy meat and it is spoiled, I will return there where I bought it and tell him “the meat that you sold to me …” I will even carry it in a sufuria and he will not refuse, he will say “ohh I remember.” And he will give me another” (FGD_Women_Kenya)*

Participants noted that purchasing primarily from one vendor also allowed the vendor to perceive the customer's loyalty and this was important in extending food on credit. Also, judging vendor trustworthiness was possible by assessing cleanliness of environment and food safety (e.g., stamps on inspected meat) attributes. Trust could be broken if scales were inaccurate or tampered with, or when there was uncertainty about the actual meat being purchased, especially when vendors included precut pieces of meat despite requests for specific parts.

In Tanzania, participants described how individuals within the community depended on each other during food shortages. Trust, and the expectations that come with it, were reciprocal.

## 4 Discussion

Basic human values drive food choice decision-making for Kenyan and Tanzanian study participants (Schwartz et al., 2012). In this study, both values with a social focus (e.g., conservation and self-transcendence), in which the food choice outcome of a social group or prevailing institutions (e.g., families) and values with a personal focus (e.g., openness to change, self-enhancement), where food choices were used to express the agency or capability of the decision-maker, were important drivers of food choice.

Prior studies that examined relationships between basic human values and food choice decision-making in higher resource settings have yielded similar findings. In a study in Greece, food choices based on convenience were motivated by values of self-direction and stimulation and conflicted with conservation and self-transcendence (Botonaki & Mattas, 2010). Chinese consumers' choices of beverages were influenced by values of security, hedonism, benevolence, and self-direction (Lee, Lusk, Mirosa, & Oey, 2014). In Japan, consumer preferences for beef complying with animal welfare and sustainability standards were influenced by the values of openness to change, self-enhancement and security (Sonoda et al., 2018). Lastly, In the Netherlands, the value of power influenced individuals' attitudes towards purchasing genetically modified foods whereas universalism influenced individuals’ attitudes towards purchasing organic foods (Dreezens et al., 2005).

The values of conservation and self-transcendence undergirded food choice in both Kenya and Tanzania. The conservation value of tradition was relevant in decisions around intrahousehold food allocation. Gender-related food traditions were concordant with existing literature on food norms, including the prioritizing of male heads of households over children (Gittelsohn, 1991; McNamara & Wood, 2019). For example, in Tanzania, intrahousehold food distribution differed by gender with only males allowed to eat certain cuts of an animal. There were various food taboos that also were related to gender, including food restrictions placed upon pregnant women and children of certain ages. Such restrictions could have the potential to deprive populations of nutritious foods (e.g., restricting eggs to children, which is a substantial source of protein and other nutrients) (Chakona & Shackleton, 2019; Ramulondi, de Wet, & Ntuli, 2021).

Changes in livelihoods contributed to a re-prioritization of tradition and concept of security in food choice decision-making. In the traditional hub model of pastoralism, women remain at the homestead near a town center and men migrate with the animals for grazing lands and water. As many adult men migrate to cities in search of better opportunities, women remain in villages to tend to the children and care for the household, but also become the head of the household, chief food choice decision-maker, and income earner. In Tanzania, traditions were described as a source of tension between the older and younger generation due to modernization which included changes in religious and educational beliefs. The value of security of the family was evident but tensions existed between financial security and procurement of nutritious foods, which were often more costly. Studies of low-income families show that they seek to preserve order in household management and avoid the chaos that can be brought about by spending outside of the budget or through a health crisis. In situations of deprivation, the value of personal and family security will be prioritized in food choice decision-making (McIntyre et al., 2003; Wiig Dammann & Smith, 2009).

Personally focused values of self-directed thought and action influenced mothers' food choice behaviors, including meal preparation decisions. Food was also an important part of seeking pleasure, novelty, and excitement. The modernization of food environments facilitated greater opportunities for indulgence, which, in turn, increased demand for highly palatable, processed foods (Vandevijvere et al., 2019; Wertheim-Heck & Raneri, 2020). Individuals who exhibit an openness to change may be more inclined to try newer or nontraditional foods and break monotony through the occasional dietary indulgence. Adults and elders also described how their children's exposure to new foods has helped inform the entire family's experience (Drew, Blake, Reyes, Gonzalez, & Monterrosa, 2023). This is consistent with a recent study in Vietnam which found intergenerational dietary shifts in the household were driven by children and adolescents, who requested preparation of nontraditional food items that they found novel and exciting (Wertheim-Heck & Raneri, 2020).

As individuals in LMICs increasingly gain access to novel highly palatable foods, further studies should investigate how values are prioritized (Andreasen, 1994; Da Silva & Mazzon, 2016; Rundle-Thiele et al., 2019). Youth in both settings were willing to try new foods and cooking methods through increased intercultural exposure at work, school or through the media. Their interest in experiencing new foods reflects the values of openness to change and stimulation. Elders valued traditional foods, often lamenting about how traditional eating practices have been largely ignored over time. Such changes were described consistently across settings and by both men and women. Policies and interventions designed to promote sustainable healthy diets could be more effective by incorporating understanding of value prioritization in different age groups and genders.

The values of achievement, face, and power undergirded food choice decision-making in this study. Participants were keenly aware of how food denoted class hierarchy, relative wealth or having desirable livelihoods. Food as a symbol of power is well documented (Jones, 2007). In both Kenya and Tanzania, livestock ownership was a symbol of prestige while meat and ultra-processed food consumption were signs of wealth and power. Given the connections between ultra-processed food, meat consumption and chronic disease, their growing consumption is of concern in LMICs (Popkin et al., 2012; Vandevijvere et al., 2019). As chronic diseases such as type 2 diabetes (Vorster, Kruger, & Margetts, 2011), cardiovascular disease (Keates, Mocumbi, Ntsekhe, Sliwa, & Stewart, 2017), cancer (Bahnassy, Abdellateif, & Zekri, 2020), and kidney disease (Kaze, Ilori, Jaar, & Echouffo-Tcheugui, 2018) continue to skyrocket in Africa, there is a growing need to promote healthy sustainable eating patterns. In the last 15 years, the rising middle class in East Africa has bought more disposable income to families that once experienced poverty (Chikweche, Lappeman, & Egan, 2021). How this new middle-class values hierarchy and symbolism of status foods will be critical to achieving healthy diets and better health outcomes. Our data suggest that younger members of the community who are more exposed to information and global trends are deviating from these ways of defining status. It is possible that an educated, wealthier new generation might ascribe different foods and ways of eating as symbols of power or success. A better understanding of values of this emerging middle class would provide valuable insight for future policy and program action.

Understanding how values, exogenous shocks, and food environments interact to drive food choices is important for future efforts to promote sustainable healthy diets. In the Tanzania study, the deleterious effects of climate change on livestock viability and agricultural productivity were repeatedly expressed among participants as a reason for some of the dietary shifts and livelihood changes. Recently, economic shocks related to the COVID-19 pandemic have been implicated in changing dietary habits (Amewu, Asante, Pauw, & Thurlow, 2020; Danquah, Schotte, & Sen, 2020; Gundersen, Hake, Dewey, & Engelhard, 2020; Pellegrini et al., 2020). Understanding the ways in which unforeseen circumstances lead people to re-prioritize their values in food choice is important for policymaking, program planning, and development of culturally relevant nutrition interventions.

Participants in Kenya spoke about how they would purchase more expensive foods (e.g., quality cuts of ASF) when they are having visitors. Though making these purchases were well outside of their regular household budget, they viewed it as an investment toward enhancing their status and social and cultural capital within their social networks, as well as preventing ridicule or disdain from others within their community. Both Bourdieu (1986) and Moore and Kawachi (2017) frame social and cultural capital as less tangible phenomena in which individuals and groups have access to emotional and social support systems in their lives that are situated within their social networks (Bourdieu, 1986; Moore & Kawachi, 2017). One's social network is largely built around their immediate environment and people who live in comparable situations (Moore & Kawachi, 2017). For both social and cultural capital, a key assumption made is that one can gain access to more tangible resources that could potentially improve their health through their social networks that they could not get otherwise (Bourdieu, 1986). The prioritization of the value of face in this case is consistent with the value of personal security, given that social capital might increase security of one's family. Such insights have important implications for understanding participants response to policies and interventions designed to increase purchase and consumption of sustainable healthy diets.

The development and implementation of agriculture, nutrition, and health policies to encourage healthier food consumption can be strengthened by an understanding of values (Cuj, Grabinsky, & Yates-Doerr, 2021). Understanding the values in specific communities or population groups often provides a starting point by which advocacy groups and thought leaders (e.g., tribal elders, NGOs, community leaders, clergy, influential people) can frame the critical issues around nutrition and health, which influence policymakers to implement policies that can benefit the population (Cullerton, Donnet, Lee, & Gallegos, 2016). All people consider characteristics of food like cost, taste, convenience and health when making food choices. It is basic human values and their prioritization that inform how these considerations are defined that drive food choice behavior. Policies to promote healthy sustainable diets that are framed around basic human values would likely be better accepted by communities (Pradeilles et al., 2019; Pradeilles, Rousham, Norris, Kesten, & Griffiths, 2016). This work provides evidence to support use of tools to assess basic human values as drivers of food choice to guide policy development and implementation efforts. Application of these findings to modification of the existing Schwartz basic human values survey is warranted to provide the tools for scaling of these findings.

Understanding values is also essential for designing successful social marketing campaigns, which use commercial marketing strategies to try to increase the acceptability of a social idea or practice among specific groups (Aceves-Martins et al., 2016). In the last decade, social marketing has been used in public health campaigns related to improving reproductive health and promoting smoking cessation in LMICs (Schmidtke, Kubacki, & Rundle-Thiele, 2021). Recently, the Schwartz human values framework has been applied for climate change social marketing campaigns (Corner et al., 2015). With respect to healthy food promotion in LMICs, these techniques are in exploratory phases (Doustmohammadian & Bazhan, 2021). Aligning health messages with prioritization of basic human values in target populations may lead to more successful and sustainable outcomes (Grier & Bryant, 2005).

This analysis used data from two studies implemented in different countries in East Africa about values that drive food choice behaviors. The focus group transcripts that were analyzed in the current study, although not representative of the Kenyan and Tanzanian populations, provide insight into a range of food-related experiences and behaviors that are common to the general populations in these settings. The focus group guides for the two countries posed different questions, limiting the extent of conclusions regarding differences between settings. The focus groups did not explicitly address values, but participants were asked to describe their food choice behaviors and to reflect on the drivers of their own and others’ food choice behaviors. The expression of basic human values driving food choice behaviors across all focus group discussions highlights the fundamental importance of this psychosocial construct. Future studies that explicitly measure the basic human values driving food choice behavior across contexts are warranted.

To our knowledge, no study has applied the Schwartz theory of basic human values to understand how values undergird food choice in LMICs. The processes through which values are prioritized and re-prioritized remain largely unknown and can be dynamic. Understanding the basic values driving food choice behavior--beyond considerations of cost, taste, convenience, and health--provides greater and more applicable information than other more individualized measures such as knowledge, attitudes, beliefs. Values are developed and shared within social groups, provide insight into collective norms and expectations for behavior, and are not reflections of only individual experience. Information about values that shape food choice behavior is essential for the design, implementation, and evaluation of policies and programs that propose to achieve changes in food choice behavior. Our study demonstrates that in communities experiencing environmental, social, and economic changes with increasing access to highly palatable foods, basic human values move beyond consideration of survival such that other values become more influential. Understanding what these values are and how these values are prioritized in food choice decision-making is essential for designing culturally relevant interventions and policies to increase demand for healthy, sustainable diets in LMICs.

## Author contributions

PDS, SB, MN, and AWG led the FGD data collection in Kenya and Tanzania. CB, EM, PDS, SB, and MN conceptualized the design of the secondary data analysis. CB led the analysis, interpretation and writing of the manuscript with contributions from all co-authors, KR, AK, LR, and SDD. And EI conducted the secondary analysis of the FGD data. All authors contributed to the interpretation of findings and reviewed drafts of manuscripts.

## Funding

This research has been funded by the Drivers of Food Choice (DFC) Competitive Grants Programs, funded by the United Kingdom Government's Foreign, Commonwealth & Development Office, and the 10.13039/100000865Bill & Melinda Gates Foundation INV-008124, and managed by the 10.13039/100008899University of South Carolina, Arnold School of Public Health, United States of America. The views expressed do not necessarily reflect the United Kingdom Government's official policies. The funders did not influence the design, implementation, analysis, or interpretation of the data presented in this manuscript.

## Ethical statement

The data collected for this analysis received institutional and country approvals for human subject research. The Tanzania study received ethics approval from Emory University Institutional Review Board (Atlanta, USA), Sokoine University (Morogoro, Tanzania), and the Tanzania Ministry of Health. The Kenya study received ethics approval from the London School of Hygiene and Tropical Medicine Research Ethics Committee (London, UK) and the International Livestock Research Institute Research Ethics Committee (Nairobi, Kenya).

## Declaration of competing interest

Declarations of interest: None.

## Data Availability

Data will be made available on request.
